# Evolution and Distribution of Saxitoxin Biosynthesis in Dinoflagellates

**DOI:** 10.3390/md11082814

**Published:** 2013-08-08

**Authors:** Russell J. S. Orr, Anke Stüken, Shauna A. Murray, Kjetill S. Jakobsen

**Affiliations:** 1 Microbial Evolution Research Group (MERG), Department of Biosciences, University of Oslo, PO Box 1066 Blindern, Oslo 0316, Norway; E-Mails: russell.orr@ibv.uio.no (R.J.S.O.); anke.stueken@ibv.uio.no (A.S.); 2 Centre for Ecological and Evolutionary Synthesis (CEES), Department of Biosciences, University of Oslo, PO Box 1066 Blindern, Oslo 0316, Norway; 3 Plant Functional Biology and Climate Change Cluster (C3), University of Technology, Sydney, PO Box 123 Broadway, NSW 2007, Australia; E-Mail: shauna.murray@uts.edu.au; 4 Sydney Institute of Marine Science, Mosman, NSW 2088, Australia

**Keywords:** cyanobacteria, dinoflagellates, harmful algal blooms (HABs), horizontal gene transfer (HGT), phylogeny, paralytic shellfish poisoning (PSP), paralytic shellfish toxin (PST), saxitoxin, STX

## Abstract

Numerous species of marine dinoflagellates synthesize the potent environmental neurotoxic alkaloid, saxitoxin, the agent of the human illness, paralytic shellfish poisoning. In addition, certain freshwater species of cyanobacteria also synthesize the same toxic compound, with the biosynthetic pathway and genes responsible being recently reported. Three theories have been postulated to explain the origin of saxitoxin in dinoflagellates: The production of saxitoxin by co-cultured bacteria rather than the dinoflagellates themselves, convergent evolution within both dinoflagellates and bacteria and horizontal gene transfer between dinoflagellates and bacteria. The discovery of cyanobacterial saxitoxin homologs in dinoflagellates has enabled us for the first time to evaluate these theories. Here, we review the distribution of saxitoxin within the dinoflagellates and our knowledge of its genetic basis to determine the likely evolutionary origins of this potent neurotoxin.

## 1. Introduction

### 1.1. Harmful Algal Blooms and Saxitoxin-Synthesis in Two Kingdoms of Life

**Figure 1:**
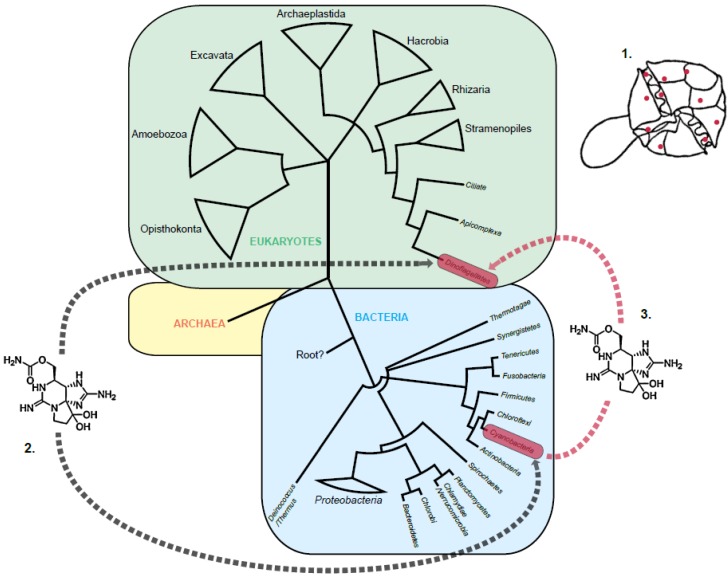
The three theories of saxitoxin (STX) evolution in dinoflagellates: (**1**) Co-cultured bacteria: Intracellular bacteria associated with the dinoflagellate cell are synthesizing STX; (**2**) Convergent evolution: The independent evolution of an analogous STX product in cyanobacteria and dinoflagellates (black dotted arrows); (**3**) Horizontal gene transfer (HGT): STX evolved in dinoflagellates after a single HGT event with cyanobacteria (red dotted arrow). The dinoflagellate and cyanobacterial lineages are highlighted in red rectangles. For illustrative purposes, the Kingdom Archaea, is collapsed. Eukaryotic [8] and bacterial [9] lineages are collapsed at different taxonomic levels.

The dinoflagellates (Dinophyceae) are a phylum of flagellated protists that belong to the eukaryotic lineage, Alveolata (Figure 1). Approximately 2000 extant species are known, 85% of which occupy marine habitats [1]. Of these, fewer than 100 have been identified as toxin producers [2]. Saxitoxin (STX) and its documented 58 analogues are environmental neurotoxic alkaloids, responsible for the human illness, paralytic shellfish poisoning (PSP) [3]. STX ingested via shellfish vectors reversibly binds voltage-gated Na^+^ and Ca^2+^ channels, blocking the neural pore, sometimes with fatal consequences [4,5]. Worldwide, PSP affects some 2000 people annually, with a 15% mortality rate [6]. This has resulted in the estimated annual global cost of US $895 million, used for monitoring and mitigation [7]. 

The gonyaulacoid dinoflagellates of the genus, *Alexandrium* and *Pyrodinium*, as well as a single gymnodinoid species, *Gymnodinium catenatum*, are known to synthesize STX [6]. *Pyrodinium bahamense* is the main STX producer in tropical waters, whilst *G. catenatum* is reported from the coasts of all continents [10,11]. *Alexandrium* spp., however, are the most abundant and widespread producer of STX [12]. Eight of the 30 known species within the genus are documented to synthesize STX [13]. Additionally, STX production within *Alexandrium* appears paraphyletic, and there are seemingly toxic and non-toxic strains of the same species [14,15]. 

In addition to marine dinoflagellates, certain freshwater cyanobacteria species (Figure 1) also synthesize STX [16]. The evolutionary history that resulted in the curious cross-kingdom distribution of STX-synthesis remained unclear. However, three theories have been proposed in an attempt to explain this pattern (Figure 1):

1. Autonomous STX-production by symbiotic bacteria associated with the dinoflagellate cell (co-cultured bacteria) [17];
2. Convergent evolution [13]; or
3. Horizontal gene transfer [18].

### 1.2. The STX Genes in Cyanobacteria and Dinoflagellates

The biosynthetic pathway and genes responsible for STX-synthesis have recently been reported in the cyanobacterial genera, *Anabaena*, *Aphanizomenon*, *Cylindrospermopsis* and *Raphidiopsis*, from the order, Nostocales, and *Lyngbya wollei* from the order, Oscillatoriales [16]. Cyanobacteria diverged some 3500 Ma [19], based on fossil evidence, with the *sxt* gene cluster believed to have emerged early in the divergence of the Nostocales, at least 2100 Ma [16]. Furthermore, within cyanobacteria, STX synthesis appears paraphyletic, as both STX producing (STX^+^) and STX non-producing (STX^−^) strains of the same species can be found [16]. This has led to the proposal that toxicity is an ancestral trait, secondarily lost for some descendants [20]. A complement of 14 genes (*sxtA*–*sxtI*, *sxtP*–*sxtS* and *sxtU*) is common between the STX-pathways of the five cyanobacterial genera [16]. These have been defined as “core” genes [3,16]. Eight of these genes (*sxtA*, *sxtB*, *sxtD*, *sxtG*, *sxtH*/*T*, *sxtI*, *sxtS* and *sxtU*) seem to be directly implicated in STX-synthesis [21]. The majority of these have seemingly originated in cyanobacteria via horizontal gene transfers (HGTs) from other bacteria, in particular, Proteobacteria [20].

In contrast to cyanobacteria, the genes involved in STX-synthesis in dinoflagellates have, until recently, remained elusive [22,23]. Dinoflagellates have large genomes, up to 60-times the size of the human haploid genome, a considerable number of unknown genes and a high frequency of repeats, making genomic studies challenging (see [24] and references therein). However, the complex and unique STX biosynthesis pathway is seemingly conserved between cyanobacteria and dinoflagellates: It involves arginine, *S*-adenosylmethionine (SAM) synthetase and acetate, with the integration of the methyl group of SAM into the final molecule [25]. To identify dinoflagellate STX genes, dinoflagellate homologues of SAM have been targeted by degenerate polymerase chain reaction (PCR) primer approaches [26]. Additionally, enzymes have been purified in conjunction with active assays [18], and transcripts have been analysed *in silico* [14,27]. However, the above approaches were unsuccessful.

Stüken *et al*. (2011) sequenced >1.2 × 10^6^ mRNA transcripts from two STX^+^ *Alexandrium* strains and demonstrated that cyanobacterial *sxt* gene homologs were transcribed in dinoflagellates [22]. The result was supported by a recent transcriptome analysis of an additional STX^+^ *Alexandrium* strain [23]. Further, Stüken *et al*. (2011) characterized the unique starting gene of STX-synthesis, *sxtA*, showing a comparable domain structure to its cyanobacterial homologue [22]. However, in contrast to the bacterial homologues, the dinoflagellate mRNA transcripts were monocistronic, had a higher GC content, occurred in multiple copies and contained typical dinoflagellate spliced-leader (SL) sequences and eukaryotic polyA-tails. The result clearly showed that dinoflagellates were autonomously synthesizing STX in their nuclear genome. The subsequent characterization of *sxtG*, the second “core” gene in the STX pathway, reinforced the original result [28]. 

The identification and characterization of STX pathway genes in dinoflagellates [22,23,28] now allows for the original three theories to be re-evaluated. In this review paper, we discuss and conclude as to which theory of STX evolution in dinoflagellates is most likely. 

## 2. Re-Evaluation of the Three Theories of STX Evolution in Dinoflagellates

### 2.1. Co-Cultured Bacteria

It has been proposed that co-cultured intracellular Actinobacteria and Proteobacteria associated with toxic dinoflagellates (Figure 1) were responsible for STX production [17,29]. This hypothesis has been investigated by numerous studies, though the results were conflicting [30,31,32,33,34]. Additionally, some of the methods used to measure bacterial metabolites have subsequently been shown to lack specificity, with compounds thought to be STX later being identified as chemical imposters [32,34]. 

Support for a co-cultured bacteria theory lessened when the structure of STX transcripts was characterized, confirming that the genes are encoded in the nuclear genomes of dinoflagellates [22,28]. Nevertheless, bacteria may still play an important role in modulating the biosynthesis of STX in dinoflagellates [35,36]. 

### 2.2. Convergent Evolution

Convergent evolution describes the independent evolution of the same biological trait in unrelated lineages. This process has been described in detail for genes involved in secondary metabolite synthesis in plants [37]. However, convergence of gene analogous usually occurs within phyla, for lineages that occupy comparable ecological niches, exposed to equivalent evolutionary pressures. For protist lineages, most examples of convergent evolution are associated with morphology, though some molecular examples exist [38]. For example, nuclear gene expression in kinetoplastids and dinoflagellates is regulated using a process of polycistronic mRNA processing involving spliced leaders [38]. As STX biosynthesis has a proposed polyphyletic origin [13], it has been postulated that the synthesis of toxins arose independently in the dinoflagellate and cyanobacteria lineages, converging on the same product (Figure 1). 

The domain structures of the genes, *sxtA* [22] and *sxtG* [28], have been shown to be shared between cyanobacteria and dinoflagellates. Convergent evolution of domain architectures is rare, with 0.4%–4% of sequences estimated to be involved in such events [39], suggesting that convergence is unlikely to have occurred. If convergent evolution was expected, lack of sequence homology may additionally be anticipated; however, multiple *sxt* homologues with high sequence identity are confirmed [22,28]. Convergence of gene analogues usually occurs within phyla, and to date, there are no examples to support such an event between lineages lacking common ancestry [38]. Further, convergence evolves from environmental pressure as a necessity to fitness or survival [40]. The ecophysiological role of STX remains uncertain, though hypotheses have suggested it as a chemical defence mechanism, cellular nitrogen storage, involved in DNA metabolism, or even chemical signalling [16]. Presently, however, limited evidence suggests that PSP toxins confer an evolutionary or survival advantage to the producer, and thus, no particular evolutionary pressure has been confirmed. The combination of these factors renders a convergent evolution of STX unlikely. 

### 2.3. Horizontal Gene Transfer

The evolution of bacterial genomes by the transfer of genes between species is well established [41]. More recently, HGT has been suggested as an important contributor in the evolution of multicellular eukaryote genomes, as well [42]. 

In relation to STX, it now seems that the majority of genes directly involved in STX-synthesis in cyanobacteria have an origin in other bacterial genomes [20]. The STX pathway in dinoflagellates may have also spread via a HGT process, and a single cyanobacterial origin has been postulated [18]. Indeed, dinoflagellate nuclear genomes have been shown to be highly chimeric. Multiple endosymbiotic gene transfers (EGTs) have contributed genes primarily of plastid origin [43], whereas HGTs have supplied genes of prokaryotic origin [42]. For example, the dinoflagellate histone-like proteins and form II RuBisCO were both acquired from Proteobacteria [27,44,45]. Additionally, recent work shows that mycosporine-like synthesizing genes have been transferred from cyanobacteria to dinoflagellates [46]. Thus, it is plausible that the *sxt*-homologues identified in dinoflagellates have been introduced via HGT.

## 3. The Source of STX in Dinoflagellates

Stüken *et al*. (2011) proposed that the STX gene cluster in dinoflagellates might have occurred by way of an HGT event, with an ancestral STX-producing bacterium as the source [22]. However, caution was taken in defining which bacterium was responsible, with results being somewhat inconclusive [22]. HGTs have supplied dinoflagellates with genes from both cyanobacterial [46] and proteobacterial sources [27,44,45], and in the following section, we will discuss the factors supporting both as the possible source of dinoflagellate STX. 

Of the 14 “core” STX genes (*sxtA*–*sxtI*, *sxtP*–*sxtS* and *sxtU*) common between five cyanobacterial STX^+^ strains [3,16], dinoflagellate homologues, or candidate genes, are presently identified with 10 of these (*sxtA*, *sxtB*, *sxtD*, *sxtF–I*, *sxtQ*, *sxtS* and *sxtU*) [22,23]. However, with varying degrees of significance inferred between sequences, the number of “true functional” *sxt* homologues is probably fewer. The result could be interpreted as indicating that some *sxt* gene transcripts are yet to be sequenced, though Hackett *et al*. (2013) estimated a 92% coverage of their *Alexandrium* transcriptome [23]. Sequence conservation might be so low that reliable homologue identification is impossible or, if several homologues are indeed missing in the dinoflagellates, alternative dinoflagellate genes could have substituted their functions in the SXT pathway. Alternatively, it could imply the independent evolution of the STX biosynthetic pathway in cyanobacteria and dinoflagellates [23].

### 3.1. A Proteobacterial Source of STX

Half the genes that seem to be directly implicated in STX-synthesis in cyanobacteria, which include the three initial pathway genes (*sxtA*, *sxtG* and *sxtB*), have their origin from proteobacterial genomes [20]. Additional genes indicate that the pathway was later assembled in the cyanobacterial ancestor [20]. Correspondingly, the three initial pathway gene homologues in dinoflagellates have an apparent proteobacterial origin, with no homologues being acquired directly from cyanobacteria [22,23,28]. In total, half of the dinoflagellate STX homologues identified to date have a putative proteobacterial origin, suggesting this as the probable HGT source. However, no Proteobacteria strains have been shown to synthesize PSTs at present. Proteobacteria, intriguingly, have already been shown to have a tight association with STX^+^ dinoflagellate strains [30,31,32,33,34], previously causing suspicion that they were responsible for STX production [17,29,47]. Further, acquisition of novel genes from endosymbionts via HGT has been highlighted to modulate the evolution of eukaryotic genomes [48].

### 3.2. A Cyanobacterial Source of STX

It is interesting that the three initial pathway genes, possibly the most important in STX-synthesis, have an apparent shared proteobacterial origin in both cyanobacteria and dinoflagellates [20,22,23]. Further, *sxtA*, the unique starting gene of STX-synthesis in cyanobacteria, is a possible result of two independent HGTs, one from an actinobacterial species and another from a Proteobacterium, followed by gene fusion [20]. This again is mirrored in the dinoflagellate homologue, where the A1 (SAM-dependent methyltransferase) and A4 (class II aminotransferase) domains of *sxtA* have a comparable phylogenetic history [22]. The result may indicate that the STX pathway in dinoflagellates was acquired by an HGT with a cyanobacterium, as was originally postulated prior to the finding of *sxt* genes in dinoflagellates [18]. The more conserved evolutionary structure, function and sequence of the initial pathway genes might further suggest them as essential to STX-synthesis [16,22,23,28]. 

The phylogenetic inference of *sxtG* [28] revealed that a second unrelated dinoflagellate amidinotransferase has an affinity to cyanobacterial cylindrospermopsin *AoaA*/*CyrA* sequences [49]. Consequently, it was proposed that multiple amidinotransferases might have been acquired by HGT in parallel or by separate events. However, the presence of multiple homologues of cyanobacterial toxin genes in dinoflagellates may equally indicate a massive transfer of toxin-related genes from cyanobacteria to dinoflagellates. This has been further supported by the finding of genes of cyanobacterial origin in a recent work on the dinoflagellate, *Heterocapsa* [50].

### 3.3. Multiple Sources of STX

Presently, it is difficult to conclude if a single or multiple independent HGT events supplied dinoflagellates with all the *sxt* homologs needed for STX-synthesis. The results may indicate that at least the initial three genes (*sxtA*, *sxtG* and *sxtB*) have a primary origin in Proteobacteria before being transferred to a cyanobacterium and, further, to dinoflagellates in a single event [20,22,23]. Equally, as the gene organization in Proteobacteria is currently unknown, separate independent origins for cyanobacteria and dinoflagellates cannot be discounted. Homology between cyanobacteria and dinoflagellates for the remaining *sxt* genes appears low, with poor phylogenetic resolution making interpretation difficult [23]. This may otherwise suggest that the additional pathway genes have been independently acquired and assembled in the genomes of dinoflagellates [20]. Considering the combined results of Stüken *et al*. (2011), Hackett *et al*. (2013) and Orr *et al*. (2013), multiple independent origins from Actinobacteria, Proteobacteria and Planctomycetes seem plausible [22,23,28]. 

## 4. The Distribution of STX in Dinoflagellates

**Figure 2:**
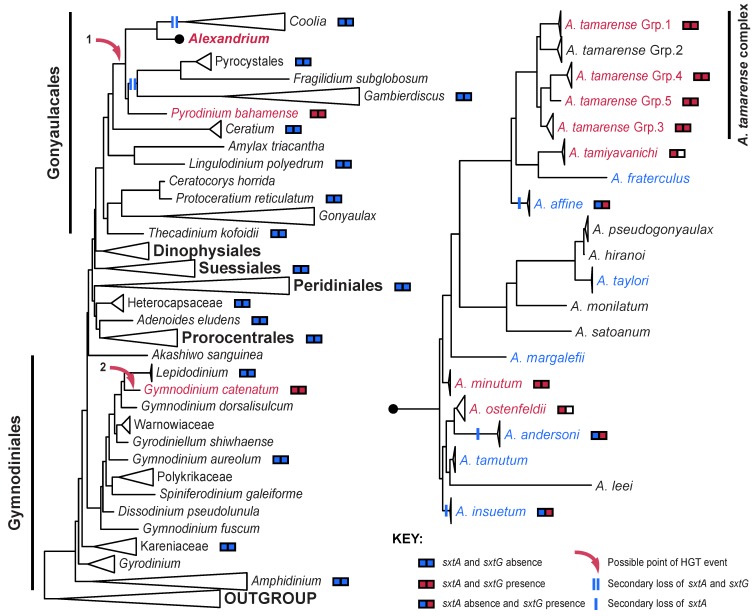
The evolution of the dinoflagellates, *Alexandrium*, and the distribution of STX, our present understanding: Only the “core” dinoflagellates are represented (to the left), with clades collapsed at different taxonomic levels [51]. The *Alexandrium* genus [15] is expanded (to the right). Red font: STX^+^ species. Blue font: STX^−^ *Alexandrium* species. *SxtA* [22,23,51,55,56] and *sxtG* [23,28] presence or absence is highlighted to the right of the name. The *sxtG* box for *A. ostenfeldii* and *A. tamiyavanichi* is blank, as they remain to be tested [55,56]. The HGT events are defined “1” and “2”, representing a primary and secondary HGT/EGT, respectively.

The primary HGT event or events that supplied dinoflagellates with the STX pathway genes is/are likely to have occurred before *Alexandrium* and *Pyrodinium* diverged within the order, Gonyaulacales (Figure 2). Considering the distribution of *sxtA* [22,51] and *sxtG* [28], any proposed HGT event deeper within the Gonyaulacales seems unlikely. However, it cannot be fully discounted until the distribution of all *sxt* homologs within the phylum is known. Consequently, *Gymnodinium catenatum*, which has an *sxtA* and an *sxtG* sequence, which are conserved to and branch within the *Alexandrium* genus [22,28], probably independently acquired STX from a secondary dinoflagellate-dinoflagellate transfer (Figure 2). A secondary transfer is further supported with improved dinoflagellate phylogenetic resolution and support and the non-detection of *sxtA* and *sxtG* for multiple STX^−^ dinoflagellate species [22,28,51].

Additionally, if an HGT occurred in the common ancestor of *Alexandrium* and *Pyrodinium*, STX-synthesis may have been secondarily lost for some descendent genera. Considering the broadly sampled dinoflagellate phylogenies presented in Orr *et al*. (2012) [51], *Coolia*, *Fragilidium*, *Gambierdiscus* and Pyrocystales may all have had this capacity (Figure 2). The possible secondary loss of at least *sxtA* and *sxtG* from these lineages may indicate an ancient HGT event. Though multiple independent origins cannot be discounted, it seems unlikely considering the close phylogenetic association between *Alexandrium* and *Pyrodinium* for multiple STX homologues [23]. However, lack of *sxt* genes from possible descendent genera may alternatively suggest that an HGT occurred in either *Alexandrium* or *Pyrodinium* independently, with the other genus later acquiring STX via a secondary dinoflagellate-dinoflagellate transfer, congruent with *G. catenatum*. This, consistent with multiple independent origins, would then reject any secondary loss for *Coolia*, *Fragilidium*, *Gambierdiscus* and Pyrocystales. To conclude fully, the position of *P. bahamense* and its relationship to *Alexandrium* needs to be more robustly resolved. A sister relationship between these taxa would substantiate a single event, simultaneously rejecting any secondary loss for non-PSP lineages. 

*Alexandrium* species are the most abundant PSP producers, and results highlight that the evolution of STX remains dynamic within the genus (Figure 2): The improved resolution for the *Alexandrium* genus, in addition to toxin testing via HPLC [15,28], reveals a paraphyletic distribution of STX-synthesis, as previously suggested [14,52]. This is further supported by the distribution of the unique starting gene of STX-synthesis, *sxtA* [22,51,53]. *Alexandrium tamarense* strains previously thought as being STX^−^ were shown as STX^+^, possessing both the A1 and A4 domains of *sxtA* and producing low amounts of STX [22,53]. The results emphasize how detection limits of non-genetic based methods can give an incomplete picture when attempting to infer the evolution of STX-synthesis [54]. Recent work on *A. tamarense* Group III strains [23] and *A. ostenfeldii* strains [55] confirms that the A4 domain of *sxtA* is essential for STX-synthesis, with STX^−^ strains of these species encoding only the A1 domain.

In contrast to *sxtA*, *sxtG* is not exclusive to STX^+^ species (Figure 2), but apparently present and transcribed in all *Alexandrium* species [28], including all tested species, for which *sxtA* and STX-synthesis have not been detected [15,22,28,51]. Consistent with *sxtA* distribution [22,51], *sxtG* was not detected in STX^−^ dinoflagellate genera external to the *Alexandrium* genus, *Pyrodinium* and *Gymnodinium catenatum* [23,28]. The distribution of *sxtG* suggests that all *Alexandrium* species originally had the capacity to synthesize STX until the recent secondary loss of essential genes. The present results imply that *sxtA*, and, in particular, the A4 domain, is essential to STX-synthesis*.* Considering Figure 2, it appears that *sxtA* has been secondarily lost within the genus on multiple occasions independently, rather than from a single event. Congruently, the loss of toxicity within cyanobacteria appears to be widespread among closely related strains with common STX^+^ ancestry [20]. 

## 5. Conclusions

### 5.1. A Revised Theory of STX Evolution in Dinoflagellates

In conclusion, we propose that STX-synthesis evolved once in cyanobacteria before the initial pathway genes (*sxtA*, *sxtG* and *sxtB*) were transferred to the dinoflagellates in a massive gene transfer event. The recipient, the ancestor of *Alexandrium* and *Pyrodinium*, acquired and assembled the additional genes in the pathway from multiple prokaryotic sources, independently of cyanobacteria. The STX pathway genes were subsequently modified extensively, taking on a eukaryotic structure, in addition to multiple homologues either being lost or replaced. During the evolution of the dinoflagellates, the pathway was secondarily lost for some gonyaulacoid lineages and transferred to *G. catenatum* through a dinoflagellate-dinoflagellate EGT event. Within *Alexandrium*, genes and domains were likely re-shuffled, with s*xtA* and the A4 domain being lost for multiple species, resulting in the paraphyletic distribution we have today (Figure 2).

### 5.2. Outstanding Questions for Future Research

Recent advances have improved our understanding of the evolution and distribution of STX within dinoflagellates. However, it is difficult to conclude, as some questions remain to be addressed. The phylogenetic relationships for the Gonyaulacales order and the *Alexandrium* genus need to be more robustly resolved, with all outstanding STX distribution questions reliant on this [15,51]. Previously and recently identified *sxt* homologues need to be characterized in detail, from multiple dinoflagellate species, to resolve the evolutionary origin of STX in dinoflagellates [22,23,57], with *sxtB* being seen as a priority before the additional “core” STX genes, *sxtD*, *sxtF–I*, *sxtQ*, *sxtS* and *sxtU*. This is crucial to further corroborate the point of single or possible multiple gene transfers, in addition to the pattern of STX loss in dinoflagellate evolution: Which genes have been lost and from which lineages? Have genes been lost from lineages independently? Are genes retained and transcribed? Are there remnants in STX^−^ genomes? Did the HGT occur deeper in dinoflagellate evolution? Further, the point of a secondary dinoflagellate-dinoflagellate transfer needs confirmation through the expanded testing of possible sister taxa to *Gymnodinium catenatum*, e.g., *Gymnodinium nolleri*.

In this review, we also emphasize that a focus is needed toward prokaryotic taxa, with the inferred phylogenies and Basic Local Alignment Search Tool (BLAST) results suggesting that multiple *sxt* homologues have either an actinobacterial or proteobacterial origin [22,23]. Interestingly, it is these bacterial lineages that are associated with STX^+^ dinoflagellate strains, with the possibility that they may have been the source of an STX transfer. We suggest that co-cultured intracellular bacteria associated with both STX^+^ and STX^−^ *Alexandrium* strains should be isolated and genome sequenced, the comparison of which may identify possible roles of intracellular bacteria in the modulation of STX-synthesis in dinoflagellates.

The work on *sxtG* [28] may indicate a massive transfer of toxin-related genes from bacteria to dinoflagellates, with recent work supporting this [50]. This should be investigated further through large-scale genomic and transcriptomic studies of dinoflagellates, with subsequent gene mining.

Finally, the methods and results presented in this review open the further development of marine probes [22,28,53], which could substantially improve environmental monitoring of harmful algal bloom (HAB) species. For example, the A4 domain of *sxtA*, the only region presently demonstrated as essential to STX-synthesis [22,23,55], could be PCR targeted to identify the presence of STX^+^ dinoflagellate strains [54]. Further, this could be correlated to toxicity, via qPCR, using standards already available from the mouse assay.

## Appendix: Glossary and Definitions

Alkaloid: An organic heterocyclic secondary metabolite containing nitrogen as the key atom. Many alkaloids have potent pharmacological effects, e.g., caffeine and nicotine.
Horizontal gene transfer (HGT): Also referred to as lateral gene transfer (LGT). Refers to the movement of genetic information across mating barriers, between distantly related organisms. In contrast, vertical inheritance or gene transfer occurs between a parent and its offspring.
High performance liquid chromatography (HPLC): A chromatographic technique used to separate a mixture of compounds to its individual components. Resulting components can be identified and quantified.
Monocistronic: An mRNA that only has a single open reading frame (ORF) and is thus translated into a single protein. This is associated with translation in eukaryotes.
Monocistronic: A taxonomic grouping that contains the common ancestor and all of its decedents.
Non-Ribosomal peptide synthetase (NRPS): Synthesizes non-ribosomal peptides (NRP) independent of ribosomes and mRNA. Each NRPS synthesizes a single peptide, though they can also work in conjunction with polyketide synthases (PKSs) to give hybrid products. Modular type I PKSs and NPRSs form megasynthetases that generally follow a collinearity rule [58], where one module extends a growing acyl or peptidyl chain by one particular unit. This enables the prediction of the chemical structure of their metabolite products [58]. All hybrid NRPS/PKS investigated so far are modular enzymes [59].
Paraphyletic: A taxonomic group that includes some, but not all, of the descendants of a common ancestor.
Paraphyletic: Taxonomic grouping that has no shared common ancestry.
Polyketide: A class of secondary metabolites that contain alternating carbonyl and methylene groups via a polyketide synthase (PKS) process.
Type I polyketide synthases (PKSs): PKSs are multi-domain enzymes or enzyme complexes that synthesize polyketides, a class of secondary metabolites. PKSs are classified into three groups, with Type I PKSs being multifunctional enzymes organized into large modules. At the genomic level, modular PKSs can be encoded as single domain enzymes in dinoflagellates [24].
Secondary metabolites: Metabolic compounds that are non-essential for the primary metabolic functions of an organism. They can largely be divided into three classes: alkaloids, terpenoids and phenolics.
Spliced leader (SL) trans-splicing: A short RNA fragment from a small noncoding RNA (SL RNA) is transplanted to the 5′ end of independently transcribed pre-mRNAs yielding mature mRNAs. The process converts a polycistronic transcript into translatable monocistronic mRNAs [60].
*SxtA*: First gene of STX-synthesis. It has four catalytic domains: A SAM-dependent methyltransferase (A1), GCN5-related *N*-acetyltransferase (A2), acyl carrier protein (A3) and a class II aminotransferase (A4) [21].
*SxtG*: An amidinotransferase enzyme that encodes the second step is saxitoxin biosynthesis [21].
Toxicity: The degree to which a substance can damage/harm an organism or its parts; this is therefore a species-specific definition.
